# Early‐onset inflammatory bowel disease as a model disease to identify key regulators of immune homeostasis mechanisms

**DOI:** 10.1111/imr.12726

**Published:** 2018-12-18

**Authors:** Julia Pazmandi, Artem Kalinichenko, Rico Chandra Ardy, Kaan Boztug

**Affiliations:** ^1^ Ludwig Boltzmann Institute for Rare and Undiagnosed Diseases Vienna Austria; ^2^ CeMM Research Center for Molecular Medicine of the Austrian Academy of Sciences Vienna Austria; ^3^ Department of Pediatrics and Adolescent Medicine Medical University of Vienna Vienna Austria; ^4^ Department of Pediatrics St. Anna Kinderspital and Children's Cancer Research Institute Medical University of Vienna Vienna Austria

**Keywords:** genetics, inborn errors of immunity, inflammatory bowel disease, pathomechanisms

## Abstract

Rare, monogenetic diseases present unique models to dissect gene functions and biological pathways, concomitantly enhancing our understanding of the etiology of complex (and often more common) traits. Although inflammatory bowel disease (IBD) is a generally prototypic complex disease, it can also manifest in an early‐onset, monogenic fashion, often following Mendelian modes of inheritance. Recent advances in genomic technologies have spurred the identification of genetic defects underlying rare, very early‐onset IBD (VEO‐IBD) as a disease subgroup driven by strong genetic influence, pinpointing key players in the delicate homeostasis of the immune system in the gut and illustrating the intimate relationships between bowel inflammation, systemic immune dysregulation, and primary immunodeficiency with increased susceptibility to infections. As for other human diseases, it is likely that adult‐onset diseases may represent complex diseases integrating the effects of host genetic susceptibility and environmental triggers. Comparison of adult‐onset IBD and VEO‐IBD thus provides beautiful models to investigate the relationship between monogenic and multifactorial/polygenic diseases. This review discusses the present and novel findings regarding monogenic IBD as well as key questions and future directions of IBD research.

## 1 BACKGROUND

### 1.1 Inflammatory bowel disease (IBD)

The gastrointestinal (GI) tract is the largest lymphoid organ in the body and contains a multitude of diverse cell types including enterocytes, Goblet cells, enteroendocrine cells, Paneth cells, but also T and B cells, macrophages, dendritic cells, and innate lymphoid cells.^1^, ^2^, ^3^ Despite the fact that these cells are constantly confronted with antigens, primarily in the form of food and bacteria, immune responses in the gut are tightly regulated to maintain homeostasis. IBD refers to a heterogeneous group of diseases that present with bowel inflammation and intractable diarrhea^4^ as a result of an inappropriate inflammatory response and unbalanced crosstalk between the gut lumen and mucosal immune system. IBD is often classified according to histopathological features as Crohn's disease, ulcerative colitis, or indeterminate colitis.^5^ Adult‐onset IBD is common and generally considered a complex, multifactorial disease where a combination of factors, including host genetics and environmental factors (including the microbiome), influence disease onset.^6^, ^7^ Due to the complex nature of adult IBD, research unraveling the genetic aberrations behind this phenotype has focused on identifying genetic risk factors using genome‐wide association studies (GWAS). In the last decade, intensive research using GWAS has identified over 230 IBD‐associated loci comprising approximately 300 potentially associated genes,^8^, ^9^, ^10^, ^11^ including *NOD2*,* ATG16L1*,* IRGM*,* IL23R*,* CARD9*,* RNF186*, and *PRDM1*. Although there are only a few GWAS SNPs with evidence of biological involvement in IBD, such as missense SNPs in *NOD2* 12 and *ATG16L1,* 13 identification of such associations pinpointed crucial mechanisms such as autophagy, pattern‐recognition, Th17 involvement, and maintenance of the epithelial barrier in IBD pathogenesis.^11^ Recent efforts have focused on meta‐analysis and fine mapping of existing GWAS datasets using innovative approaches such as Bayesian analysis,^14^ as well as adding novel, valuable cohorts to identify new loci. Among newly identified loci are SNPs pointing to integrin genes^10^ *ITGA4* and *ITGB8*. Integrins are transmembrane receptors that facilitate extracellular matrix adhesion, thereby are important in the homeostasis of the epithelial barriers.

Interest in the potential common component of immune‐mediated diseases has lead to inter‐disease comparisons and identification of shared loci between IBD and other autoimmune or inflammatory conditions such as juvenile idiopathic arthritis, primary sclerosing cholangitis, psoriasis, multiple sclerosis, and ankylosing spondylitis.^15^, ^16^, ^17^ These pleiotropic loci point to shared pathways and molecular mechanisms underlying the heterogeneous immune‐mediated diseases.

Despite current advances in data collection and analysis, our understanding of SNPs outside the coding regions is still elusive. It has been shown that SNPs for autoimmune disease tend to be enriched in regulatory regions, and in differentially expressed genes, and that risk variants for autoimmune diseases show particular enrichment in active chromatin regions of immune cells.^18^, ^19^, ^20^ In addition, several efforts have been made to unravel how SNPs at a locus affect mRNA expression of genes. These efforts combining GWAS with transcriptome analysis have revealed that pinpointing the causal SNP in a haplotype block is a non‐trivial task and that many of the SNPs have a detectable effect only in a cell type‐dependent or stimulus dependent context.^21^, ^22^, ^23^

As our knowledge for non‐coding regions of the genome is growing relating SNPs to regulatory regions, as well as assaying the cell type specificity of loci, will be important goals for the future. Notably, it is unclear how the identified susceptibility loci and associated genes identified in these GWAS studies relate to the early‐onset, Mendelian form of IBD. Identification of high impact SNPs in *NOD2* that are associated with adult Crohn's disease with clear involvement in IBD pathogenesis has illustrated a genetic continuum between adult and early‐onset IBD, in contrast to the classical view of two genetically independent diseases.^24^ In this context, we can hypothesize that adult and Mendelian IBD arise as a result of a spectrum of varyingly pathogenic genetic lesions that impact common key pathways in IBD. Despite these advances, the exact relationship between adult and Mendelian IBD is still poorly understood. The lack of understanding of (adult) IBD is also reflected in the fact that there are currently only a few stratified/personalized treatment strategies despite the recent expansion of therapies based on immune modulation, mostly using monoclonal antibodies.^25^ Given these challenges, the precise mechanisms of IBD disease pathogenesis, the relationship between adult and early‐onset IBD, and the complex interplay between host genetics and environmental factors have remained partially elusive with major gaps in our understanding in the genetic processes governing IBD pathology.^8^

### 1.2 Monogenic and Mendelian IBD

Very early‐onset IBD (VEO‐IBD) denotes a subgroup of IBD patients with a disease onset before the age of 6 years.^27^ In contrast to adult IBD, VEO‐IBD is a rare disease where mutations in causal genes may be inherited in a Mendelian fashion, as illustrated by our discovery of IL10R deficiency.^26^ VEO‐IBD patients usually present with a severe clinical course including (often bloody) diarrhea and abdominal pain.^27^ Most patients with VEO‐IBD receive immunosuppressive treatment, and many patients require surgical intervention during the course of their disease.^28^ To date, there are only a handful of monogenic defects that result in a predominant IBD phenotype, including *ADAM17*,* IL10*,* IL10RA*,* IL10RB*,* GUCY2C*,* IL21*,* LRBA*,* TTC7A*, and *XIAP*.^26^, ^29^, ^30^, ^31^, ^32^, ^33^, ^34^ Identification of these gene defects have provided proof of concept for genetic diagnosis and stratified therapeutic choices, shaping our understanding of the immune system and illustrating molecular mechanisms underlying the delicate balance in keeping the homeostasis in the gut.

**Figure 1:**
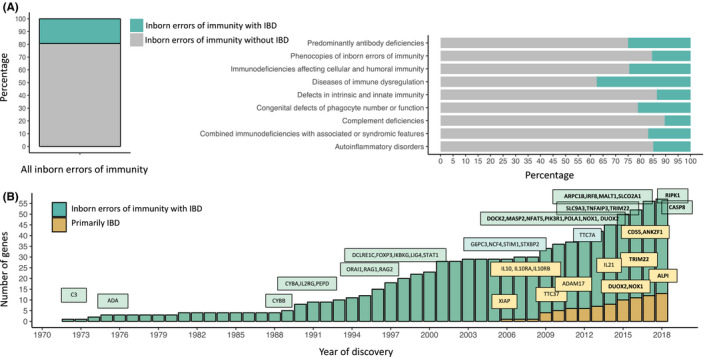
Advances in identification of genetic etiologies underlying inflammatory bowel disease and inborn errors of immunity. (A) The percentage of inborn errors of immunity with IBD. Classification according to the 2017 International Union of Immunological Societies (IUIS) phenotypic classification of inborn errors of immunity.^35^ (B) Discoveries of inborn errors of immunity with IBD and VEO‐IBD genes through the years. Gene defects that were described between 2015 to 2018 are highlighted in bold

Intriguingly, a spectrum of inborn errors of immunity (IEI) can present with an IBD‐like phenotype, sometimes as the initial disease manifestation. IEIs are a heterogeneous group of more than 330 different disorders with around 300 genes currently identified to be associated with monogenic, Mendelian forms.^35^ The main characteristics of IEIs are increased susceptibility to infections due to improper function or dysregulation of key players of the immune system. These observations highlight the interesting fact that a spectrum of different immunopathological processes can underlie GI inflammation and point to the GI tract as an exceptionally sensitive site to immune disturbances. Current consensus estimates that about 20% of genetic defects underlying IEIs can develop bowel inflammation (Figure 1A). The International Union of Immunological Societies (IUIS) recognizes 9 phenotypic groups of IEIs.^35^ Among the functional groups of IEIs, diseases of immune dysregulation present most often with an IBD‐like phenotype in up to 40% of the different genetic defects. On the other hand, complement deficiencies tend to present without bowel inflammation (95% of known gene defects do not cause IBD, Figure 1A). To date, considerably accelerated by the advent of next‐generation sequencing, >60 monogenic diseases that present with IBD have been described^27^, ^36^ (Figure 1A). Between the year 2015 and 2018 alone, several new gene defects have been identified that underlie some type of bowel inflammation (Figure 1B).

**Figure 2:**
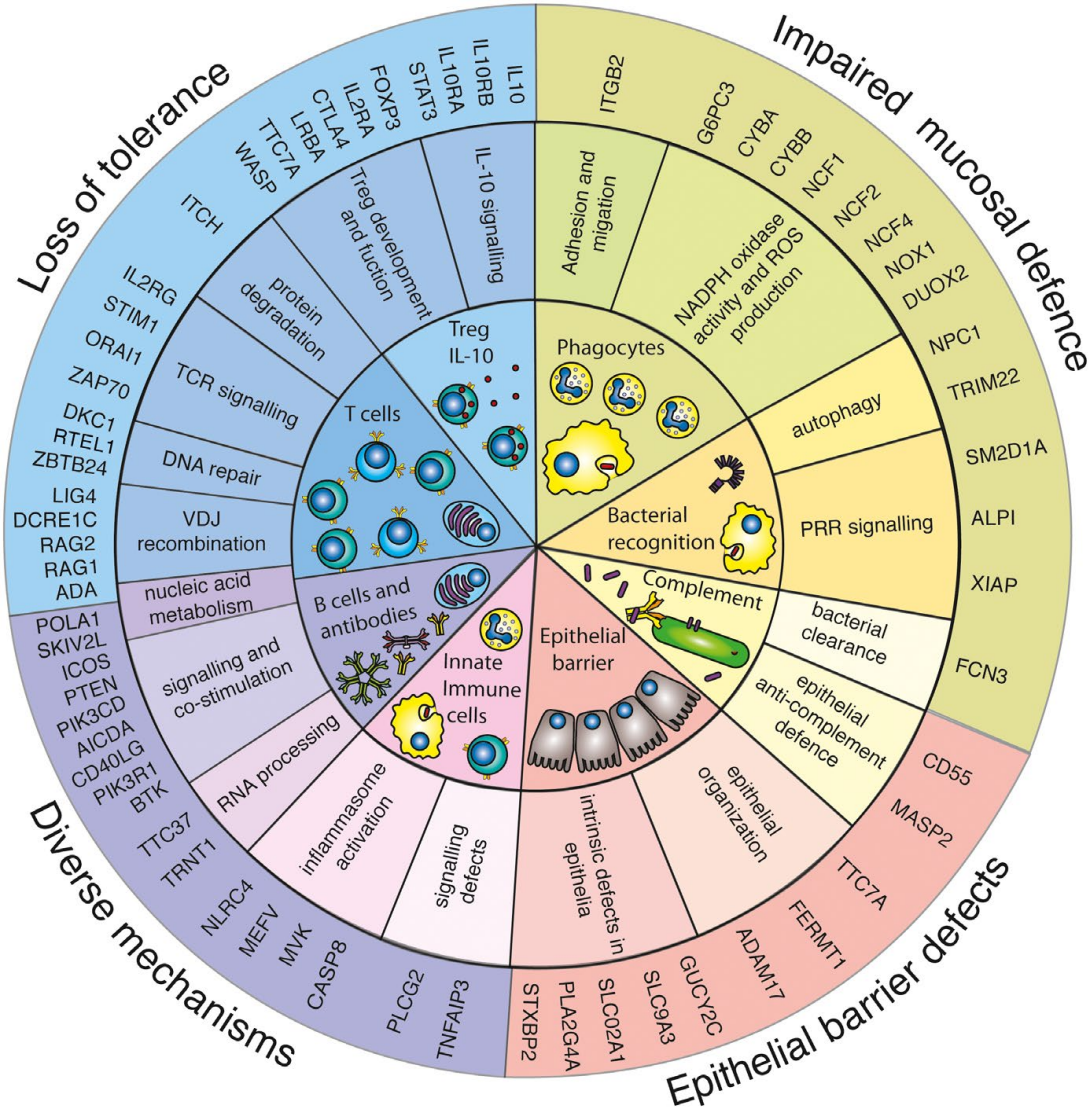
Cell types and molecular mechanisms involved in the pathogenesis of inflammatory bowel disease. The inner circle represents cell types and cell components involved in IBD pathogenesis, as detailed in the text. The middle circle depicts the molecular mechanisms affected by mutations in genes presenting with an IBD phenotype. The outer circle represents the molecular pathomechanisms leading to IBD. Treg IL10: T‐cell immunodeficiencies with bowel inflammation and Defects in Tregs or IL10 signaling. Phagocytes: Congenital defects of phagocyte number or function. Complement: Complement deficiencies. Bacterial recognition: Defects in host‐microbiota interactions, bacterial sensing. Epithelial barrier: Epithelial barrier defects. B cells and antibodies: Predominantly antibody deficiencies with IBD. Innate immune cells: Systemic autoinflammatory diseases and IBD. PRR: pattern‐recognition receptor

Interestingly, some gene defects in subgroups of IEI do present with bowel inflammation, while other gene defects in the same group do not. While currently, there is no comprehensive and satisfactory explanation for the varying frequency of the IBD phenotype in individual gene defects, one can speculate that (a) due to the few patients and therefore small sample size in rare diseases, it is possible that certain phenotypes of inborn errors of immunity have not yet been captured, especially when it comes to disease with only one patient described at present, (b) our knowledge of the explicit effects of genetic aberrations is incomplete; therefore, it is plausible that in some gene defects counter‐mechanism are in place and can maintain a pseudo‐homeostatic state in the gut, therefore not inducing an IBD‐like phenotype, and (c) since our understanding of the influence of factors extrinsic to genetic triggers is incompletely studied and understood in EO‐IBD, it is likely that (similarly to adult IBD) in some cases the EO‐IBD phenotype only emerges as a result of strong non‐genetic triggers on a genetically susceptibly host.

Investigating the consequences of genetic aberrations in patients with monogenic defects causing IBD allows for a precise dissection of genotype‐phenotype relationship. Moreover, through understanding of the mechanistic effect of pathogenic mutations on gene regulation, we have widened our knowledge on principal immune processes. Therefore identification of monogenic defects underlying IBD has not only provided genetic diagnosis to patients, but also proven to yield invaluable insights into how the immune system works. We here review monogenic defects underlying IBD and how dissection of their molecular pathophysiology has contributed to our understanding of immune homeostasis in the gut in health and disease.

## 2 MONOGENIC FORMS OF INFLAMMATORY BOWEL DISEASE

### 2.1 Epithelial barrier defects

The intestinal epithelium forms both a physical and biochemical barrier between gut microbiota and the immune cells within the mucosa. Therefore, dysregulation of the gut epithelium can result in immune overactivation that culminates in bowel inflammation. The onset of IBD can arise through the following mechanisms: (a) defects of epithelial organization, (b) defects leading to epithelial apoptosis and necroptosis, and (c) defects of epithelial‐intrinsic cellular function.

TTC7A, a member of TPR domain‐containing proteins is thought to have diverse functions in cell cycle control, protein transport, phosphate turnover, and protein trafficking and secretion. Patients with TTC7A deficiency typically present with features of severe combined immunodeficiency (SCID), along with severe exfoliative apoptotic enterocolitis.^30^, ^37^, ^38^ In previous studies, mutations in TTC7A were reported to have multiple intestinal atresias (MIA) possibly due to the constant inflammation and apoptosis of the epithelium. It appears that patients with complete loss‐of‐function typically present with MIA‐SCID phenotype, whereas milder (hypomorphic) mutations may present with EO‐IBD as a predominant phenotype.^37^, ^38^ TTC7A‐deficient patient‐derived organoids show defective apicobasal polarity and have increased apoptosis that may cause a physical breach of the epithelium therefore aggravating the bowel inflammation.^37^ However, the involvement thymic stromal‐intrinsic TTC7A deficiency in the context of T‐cell maturation and TTC7A in T‐cell intrinsic defect of activation has to be considered as potential cause for bowel inflammation in TTC7A deficiency.

In Kindler syndrome, mutations in *FERMT1* lead to lack of Kindlin 1 and an induction of inflammatory response in keratinocytes via paracrine communication. Kindlin 1 is involved in integrin signaling and the linkage of the actin cytoskeleton to the extracellular matrix. Patients with Kindler syndrome have been reported to have ulcerative colitis,^39^, ^40^, ^41^ and *Fermt1* ^−/−^ mouse model shows gut epithelial detachment due to a lack of epithelial integrin activation.^42^ This was hypothesized to cause epithelial barrier breach, which culminated in bowel inflammation in this model. Mutations in the *COL7A1* gene elicit an autoimmune response and autoantibodies to type VII collagen and cause epidermolysis bullosa dystrophica.^43^ The mutated COL7A1 leads to a deficiency in anchoring fibrils, which in turn impairs the adherence between the epidermis and the underlying dermis similarly resulting in an impaired gut epithelial barrier.

Mutations in *guanylate cyclase 2C* (*GUCY2C*), an intestinal receptor for bacterial heat‐stable enterotoxins cause relatively mild early‐onset chronic diarrhea and is associated with increased susceptibility to IBD, small‐bowel obstruction, and esophagitis.^44^ Although the exact molecular mechanism behind the familial diarrhea is yet to be determined, it has been shown that the expression of mutant GUCY2C results in increased production of cGMP, possibly underlying the hyperactivation of CFTR, leading to increased chloride and water secretion from enterocytes. Missense, splicing, and truncation mutations in *SLC9A3,* identified in nine patients from eight families lead to congenital sodium diarrhea (CSD).^45^ Two of these nine patients developed IBD at 4 and 16 years of age.^45^ SLC9A3 is an epithelial brush‐border Na/H exchanger that uses an inward sodium ion gradient to expel acids from the cell. Several members of the *SLC9A* family of Na^+^/H^+^ exchangers are expressed in the gut, with varying expression patterns and cellular localization. They participate in the regulation of basic epithelial cell functions, including control of transepithelial Na^+^ absorption, intracellular pH, cell volume, and nutrient absorption, and also in cellular proliferation, migration, and apoptosis. In addition, these proteins modulate the extracellular milieu to facilitate other nutrient absorption and to regulate the intestinal microbial microenvironment.^46^ The functional consequence of loss‐of‐function *SLC9A3* gene variants (ie, reduced sodium uptake and proton exchange at the luminal surface) appears similar to that of gain‐of‐function (GOF) variants in the *GUCY2C* gene, showcasing a potential overlapping molecular mechanism. However, the underlying mechanism of bowel inflammation in these patients is unclear. One potential hypothesis includes physical epithelial damage due to distended bowel resulting in microbiota‐mediated immune activation and bowel inflammation.

Loss‐of‐function (LOF) mutations in the *SLCO2A1* gene, encoding a prostaglandin transporter have been described to cause pediatric‐onset chronic nonspecific multiple ulcers of the small intestine, accompanied with persistent blood and protein‐losing enteropathy^47^, ^48^ in the Japanese population. Mutations in *SLCO2A1* have been previously reported as the cause of primary hypertrophic osteoarthropathy (PHO).^49^, ^50^ Three out of five male patients with chronic enteropathy associated with *SLCO2A1* had all of the major clinical features of PHO as well, such as digital clubbing, periostosis, and pachydermia. SLCO2A1, naturally expressed on the cellular membrane of vascular endothelial cells in the small intestinal mucosa, was absent from the patients’ epithelium, pointing to a potential epithelial‐intrinsic cell defect. Similarly, a LOF mutation in the *PLA2G4A* gene, encoding for cytosolic phospholipase 2‐α, has been identified in patients with cryptogenic multifocal ulcerating stenosing enteritis (CMUSE).^51^ It was shown that these patients lack protein expression in their gut epithelium. Phospholipase 2‐α is an enzyme important in the formation of prostaglandin. Together, these gene defects point toward the role of prostaglandin in gut epithelial homeostasis, specifically in the context of epithelium‐intrinsic defects. However, the exact molecular mechanism of prostaglandin‐associated enteropathy is still unclear.

Familial hemophagocytic lymphohistiocytosis (FHL) is caused by recessive mutations that impair cytotoxic function and is characterized by fever, splenomegaly, bicytopenia, high triglycerides/low fibrinogen, hemophagocytosis, high ferritin, low natural killer (NK) cell cytotoxicity, and high soluble CD25.^52^ FHL type 5 is initiated by mutations in the *STXBP2* (Munc18‐2) gene, encoding a protein involved in intracellular trafficking, the control of soluble NSF attachment protein receptor (SNARE) assembly, and the release of cytotoxic granules by NK cells.^53^ Notably, MUNC18‐2 deficiency (unlike other FHL) is often accompanied by colitis,^53^ although GI symptoms are not a common feature of FHL type 2, FHL type 3, or Griscelli Syndrome type 2 patients, suggesting that the pathology of FHL does not necessarily lead to GI disease, even in the more severe FHL subtypes. Munc18‐2 proteins have been described to have widespread expression in epithelial tissues, such as the kidney and intestines, with localization to the apical surface of the plasma membrane.^54^, ^55^ Thereby, Munc18‐2 might be essential for maintaining epithelial integrity in GI epithelial cells, but more mechanistic studies are required to determine the how Munc18‐2 deficiency lead to bowel inflammation.

### 2.2 Congenital defects of phagocyte number or function

Emerging evidence suggests that neutrophil function plays an important role in intestinal integrity, as highlighted by IBD in patients with either quantitative or qualitative neutrophil deficiencies. Neutrophil function in the gut is not restricted to the killing of bacteria that have translocated across mucosal epithelium. During the inflammatory response, neutrophils also contribute to the recruitment of other immune cells and facilitate mucosal healing by releasing mediators necessary for the resolution of inflammation.^56^ Even though our understanding of neutrophils’ role in intestinal homeostasis and their complex interactions with intestinal epithelial cells is still incomplete, gut pathologies in patients with neutrophil defects has revealed several important mechanisms.

Neutrophil nicotinamide adenine dinucleotide phosphate oxidase (NOX) is the enzyme complex responsible for generation of superoxide and other reactive oxygen species (ROS) in phagocytic cells. Mutations in the *CYBB* and *CYBA*,* NCF1*,* NFC2,* and *NCF4* genes, encoding for the cytosolic subunits of NOX, abrogate its activity and compromise host immunity against certain bacteria and fungi. These defects cause chronic granulomatous disease which are characterized by immunodeficiency and can cause IBD‐like intestinal inflammation.^57^ Inflammatory reactions in CGD patients (namely colitis) might be a result of impaired anti‐bacterial protection due to impaired NOX activity, resembling defects in ephithelial‐specific NADPH Oxidase 1 (NOX1) and Dual Oxidase 2 (DUOX2) in patients with severe EO‐IBD. Both NOX1 and DOUX2 are epithelial NADPH oxidases involved in the generation of ROS in the gut epithelium.^58^ Mutations in *NOX1* and *DUOX2* result in reduced ROS production and cause a 10‐fold increase in bacterial invasion.^59^ Impaired mucosal defense may represent a key pathomechanism that results in intestinal inflammation and development of IBD. Another possible pathomechanism leading to colitis in CGD patients is inflammmasome hyperactivation. Intriguingly, NOX‐deficient mice exhibited a skewed Th17 phenotype suggesting a possible role of pathogenic Th17 cells in development of inflammatory reactions.^60^ These data indicate that while reactive oxygen species are used by the immune system to eliminate infections they may also serve as signaling intermediates to coordinate the efforts of the innate and adaptive immune systems resulting in a complex etiology underlying phagocyte defects.

Although the exact molecular link has not been established yet, it has been shown that mononuclear phagocytes from CGD patients have increased secretion of IL‐1β that could be controlled by IL‐1 receptor antagonist (IL‐1RA) ex vivo and during treatment with anakinra.^61^

Impaired mucosal defense underlying colitis might be one of the pathomechanisms in the other types of neutropenias that result in impaired function or recruitment of neutrophils. Mutations in *G6PC3*, encoding the catalytic subunit of glucose‐6‐phosphatase (G6Pase) cause severe congenital neutropenia type IV (SCN IV) and predispose patients to IBD.^62^, ^63^, ^64^ SCN IV has been linked to glycogen storage disease type 1b as both disorders involve disruption of the glucose‐6‐phosphatase/glucose‐6‐phosphate transporter complex, leading to developmental or functional defects in neutrophils. The function of NADPH oxidase in phagocytes from patients with G6PC3 was diminished, abrogating normal ROS production.^65^ These defects suggest loss of protective function perhaps may be the main pathomechanism underlying predisposition to IBD in a subset of G6PC3‐mutant patients.

Leukocyte adhesion deficiency type 1 (LAD1) is caused by mutations in the *ITGB2* gene, an integrin participating in cell adhesion and cell surface‐mediated signaling. The disease is characterized clinically by delayed umbilical cord separation, recurrent life‐threatening infections, impaired pus formation, poor wound healing, and persistent leukocytosis. These clinical features are consequences of defective leukocyte adhesion to endothelial cells, the absence of transmigration into inflamed tissues as well as deficient phagocytosis and chemotaxis of granulocytes, monocytes, and lymphoid cells.^66^ Some patients develop an IBD‐like phenotype, most likely due to the complex pathology caused by dysregulated recruitment of leukocytes into the intestine that abrogates mucosal defense and regulation of immune response.^67^, ^68^

### 2.3 Defects in host‐microbiota interactions, bacterial sensing

Nucleotide‐binding and oligomerization domain (NOD)‐like receptors act as a first line of defense against invading bacteria. Within the NOD family, NOD2 functions as an intracellular sensor for peptidoglycans from the bacterial cell wall. NOD2 has long been studied and is recognized as a critical player in Crohn's disease pathogenesis, where it was shown to regulate innate immunity through NF‐κB‐induced proinflammatory responses.^12^ Intriguingly, single gene defects involving *NOD2* cause Blau syndrome, an inflammatory disorder phenotypically characterized by the triad of granulomatous polyarthritis, dermatitis and uveitis, however without bowel inflammation.^69^ In this context, it is postulated, that gene defects that do not directly disrupt NOD2 function, but rather de‐regulate proper NOD2 signaling, do present with IBD, whereas at least the Blau syndrome‐associated mutations in *NOD2* do not. The very first of discovery relating IBD to defective NOD2 signaling without *NOD2* mutations was XIAP deficiency.^29^, ^70^

X‐linked lymphoproliferative (XLP) disease is a rare immunodeficiency caused by mutations in the *SH2D1A/SAP* or *XIAP* genes, respectively. XLP is characterized by severe immune dysregulation that presents with susceptibility to EBV‐triggered lymphoproliferative disease (EBV‐LPD) or hemophagocytic lymphohistiocytosis (HLH), lymphoma, and dysgammaglobulinemia.^71^, ^72^ *SH2D1A* encodes the signaling lymphocyte activation molecule (SLAM)‐associated protein (SAP). SAP is involved in the function of cytotoxic lymphocytes and is a key regulator of normal immune function in T and NK cells, as well as the of NK‐cell apoptosis.^73^, ^74^, ^75^ Mutations that disrupt the SAP protein impair proper signalling to induce immune response toward viral (EBV) infection and led to the development of lymphomas due to defective lymphocytes apoptosis. Large gene deletions in the *SH2D1A* gene (up to 11 Mb) including those involving the whole gene were identified in 5 families. Three of these larger deletions were associated with GI symptoms of colitis and gastritis.^71^ XIAP plays an essential role in the regulation of apoptotic cell death induced by viral infection or an over‐production of caspases. In addition to this role, XIAP is also responsible of the regulation of RIPK2, a protein vital in NOD2 signaling. Mutations in *XIAP* cause a unique IEI, similar to X‐linked familial hemophagocytic lymphohistiocytosis and X‐linked Lymphoproliferative syndromes. Patients with *XIAP* mutations can also develop very early‐onset IBD.^71^, ^76^ The IBD phenotype in XLP2 is hypothesized to be brought on by abrogated NOD2‐mediated signalling and result in innate and adaptive immune defects including granulomatous colitis and perianal disease. Therefore, it is postulated hat colitis may be clinically and pathologically different between XLP1 and XLP2.^77^

Two additional novel gene defects that influence NOD2 signaling and present with bowel inflammation have been described recently. Mutations in the *NPC1* gene, encoding a protein that mediates intracellular cholesterol trafficking of endosomes and lysosomes, cause a neurodegenerative lysosomal storage disease, coupled with fistuling colitis with granuloma formation.^70^ The pathogenic mutations in NPC1 is thought to elicit impaired autophagy due to defective autophagosome function. Similar to XIAP deficiency, mutations in NPC1 abolishes NOD2‐mediated bacterial handling. However, *NPC1* mutations do not impair RIPK2‐XIAP dependent cytokine production.

Identification of patients with homozygous *TRIM22* mutations provided additional links of NOD2 to VEO‐IBD. TRIM22 is a ubiquitin ligase that influences NOD2 activity by ubiquitination.^78^ Mutations in *TRIM22* disrupt the ability of TRIM22 to regulate NOD2‐dependent activation of IFN‐β signaling and NFkβ. Intriguingly, LOF variants in NOD2 have been shown to result in the loss of NF‐κB‐induced proinflammatory cytokine response to muramyl dipeptide (MDP),^12^ mirroring the defects observed in patients with *TRIM22* mutation.

Expanding the spectrum of disorders of bacterial sensing underlying bowel inflammation are novel biallelic‐inherited LOF mutations in *ALPI*. ALPI is an intestinal alkaline phosphatase that is thought to function in the detoxification of lipopolysaccharide (LPS) and prevention of bacterial translocation in the gut. Mutations in *ALPI* abrogate the regulation of host‐microbiota interactions and restrain host inflammatory responses causing early‐onset severe diarrhea, weight loss, and severe ulcerations from transverse colon to the rectum.^79^

### 2.4 Predominantly antibody deficiencies with IBD

The molecular mechanisms of IBD in patients with defects in humoral immunity are not yet completely understood. Impaired antibody production, especially low IgA, may contribute to the development of gut dysbiosis, but the defects in antibody deficiency alone do not result in intestinal disease. The pathomechanism in this case is most likely due to combined T‐ and B‐cell defects.^80^ Some of the predominantly antibody deficiencies that can present with IBD include: (a) selective IgA deficiency with unknown gene defect resulting in defective B‐cell maturation into IgA‐secreting plasma cells, (b) agammaglobulinemia due to BTK or PIK3R1 deficiency leading to the lack of mature B cells and absent IgM, IgG, and IgA,^80^, ^81^ (c) X‐linked hyper IgM syndrome due to CD40LG deficiency resulting in defective co‐stimulation signaling vital for B‐cell proliferation and class‐switch,^80^ (d) activation‐induced cytidine deaminase (*AICDA*) deficiency with abrogated somatic hypermutation, gene conversion, and class‐switch recombination of immunoglobulin genes in B cells. Additionally, mutations in *PIK3CD* causing Hyper IgM syndrome (HIGM) result in intrinsic defects in both B and T cells. Clinical heterogeneity in patients with *PIK3CD* GOF mutations correlates with differences in immunological findings and suggests that development of bowel inflammation correlates with more pronounced T‐ and B‐cell defects.^82^

Three novel gene defects associated with impaired humoral immunity and gut abnormalities have recently been described. These gene defects, although all affecting humoral immunity, most likely have distinct mechanisms underlying the observed phenotypes.

Patients with PTEN Hamartoma Tumor Syndrome (PHTS) develop autoimmunity, extensive adenoid lymphoid hyperplasia requiring steroid treatment and adenotomy, thymic hyperplasia, and indeterminate colitis.^83^ PTEN is a multifunctional dual phosphatase targeting both lipid and protein targets. It mainly dephosphorylate phosphatidyl inositol‐3,4,5‐triphosphate (PIP3), an activator of PKB/Akt kinase. Therefore, PTEN is a negative regulator of the PI3K/Akt signaling. Reduced PTEN activity in PHTS affects the homeostasis of germinal centers in B cells by aberrant PI3K/Akt/mTOR pathway thereby disturbing antiapoptotic signals. Patients with heterozygous germline mutations in *PTEN* have been reported to present with B‐cells defects, including impaired class‐switching, decreased somatic hypermutation frequency and hypogammaglobulinemia.^84^ These patients show similarities to patients with GOF mutations in *PIK3CD* where B‐cell defects and increased Akt activity can be observed. Differences in clinical presentation such as hemartomas, GI polyps and lipomas not seen in PIK3CD‐mutant patients, might be explained by the broader expression pattern of PTEN.

Tricho‐hepato‐enteric syndrome (THES), also known as syndromic or phenotypic diarrhea, is a congenital enteropathy due to mutations in the *TTC37* gene. Patients with THES present with diarrhea, growth retardation, hair and facial abnormalities, and immunodeficiency. The associated malabsorption leads to malnutrition and failure to thrive. While the exact function of the *TTC37* protein is not known, some studies reported TTC37 as a component of the Ski complex which is crucial for the accurate processing of nuclear RNA precursors and degradation of both cytoplasmic and nuclear RNA.^85^ Preliminary studies of brush‐border ion transporters in enterocytes from 5 patients demonstrated their reduced expression or mislocalization.^86^ While this study suggests that the diarrhea in THES patients might be a result of intrinsic defects in enterocytes, most of the patients also develop humoral immune defects with low protective immunoglobulin (Ig) levels or poor vaccination response. A recent discovery identified a patient with TTC37 mutation, presenting with immunodeficiency but without diarrhea.^87^ While quantitative Ig concentrations were normal, response to pneumococcal vaccination was abnormal with rapid loss of protective titers, pointing to a B‐cell defect characteristic for this deficiency. It is unclear why this patient did not develop defects in GI and diarrhea, but these findings may indicate an unexpected genotype‐phenotype spectrum in this disease.

TRNT1 enzyme deficiency is a novel metabolic disease caused by defective post‐transcriptional modification of mitochondrial and cytosolic transfer RNAs. TRNT1 functions as a CCA‐adding enzyme by catalyzing the addition of the conserved nucleotide triplet CCA to the 3′ terminus of tRNA molecules. Mutations in *TRNT1* cause a complex multisystem disease, including B lymphocyte immunodeficiency and infantile‐onset cyclical aseptic febrile episodes with vomiting and diarrhea, characterized by global electrolyte imbalance during these episodes.^88^ Although the IBD phenotype is currently attributed to intrinsic defects of the gut tissues, whether defects in humoral immunity may contribute to GI inflammation is still to be investigated. Bone marrow transplantation in two patients led to encouraging results, although more long‐term data are needed to clarify the disease etiology.

Inducible T‐cell costimulatory (ICOS) is an activation‐induced member of the CD28 family on T cells. Mutations in the *ICOS* gene cause ICOS deficiency, presenting with common variable immunodeficiency (CVID) including splenomegaly, autoimmune manifestations, recurrent bacterial infections, and IBD.^89^ Absence of ICOS results in abrogation of germinal center formation leading to severe reduction of class‐switched memory B cells, as well as reduction in naïve B cells. The presumed cause of the IBD phenotype in ICOS deficiency is insufficient IL‐10 production by ICOS‐deficient T cells.^89^

### 2.5 T‐cell immunodeficiencies with bowel inflammation

Gene defects that disturb adaptive immune cell selection, activation, and differentiation can all manifest in complex immune signaling disturbances, which can result in immunodeficiency, autoimmunity, and intestinal inflammation. Monogenic gene defects underlying IEI and IBD have been essential in improving our understanding of the complex machinery of immune regulatory cascades and identified novel players in immune processes. SCID denotes a group of disorders of genetic defects that abrogate T‐cell development. Mutations in any of the genes that underlie SCID can cause an IBD‐like pathology. In particular, hypomorphic mutations where the proteins and/or molecular functions are impaired but residual activity can be observed often lead to IBD. Hypomorphic mutations in SCID‐causing genes that affect development of TCR repertoire may allow development of oligoclonal and poorly functioning T cells and are associated with a broad clinical phenotype that may include inflammatory and autoimmune manifestations, including intestinal inflammation.^90^, ^91^ Therefore, all partial T‐cell defects can potentially be associated with (severe) immune dysregulation and IBD. Here, we discuss a few examples of genetic defects in this group.

Ommen syndrome, namely impaired V(D)J recombination due to mutations in *RAG1* and *RAG2*,^92^, ^93^, ^94^ and defective DNA repair after V(D)J recombination by mutations in *DCLRE1C/ARTEMIS* 95 cause SCID characterized by erythroderma, desquamation, alopecia, eosinophilia, hepatosplenomegaly, elevated serum IgE levels, and often, colitis.^96^ Moreover, defects in *DNA ligase 4* (*LIG4*) encoding an ATP‐dependent DNA ligase that joins double stranded breaks during non‐homologous end joining pathway, and is essential for V(D)J recombination, can cause SCID, and can develop IBD.^92^

Impaired V(D)J recombination results in an emergence of an oligoclonal T‐cell repertoire, which indicates that the thymic selection in patients with Omenn syndrome is restricted to the T cell in which recombinase activity is sufficient to generate a functional TCR.^97^

Adenosine deaminase (ADA) deficiency leads to an accumulation of toxic purine degradation by‐products, most potently affecting lymphocytes, but other manifestations include skeletal abnormalities, neurodevelopmental affects, and pulmonary manifestations associated with pulmonary‐alveolar proteinosis.^98^ The major consequences of *ADA* mutations are severe depletion of T and B lymphocytes and NK cells. The underlying mechanisms of this deleterious effect are the increased apoptosis due to the buildup of dATP in cells especially in developing thymocytes and T cells.^99^ Although patients present with severe B‐lymphocytopaenia and hypogammaglobulinaemia, B‐cell development seems to be unaffected.^100^

Interleukin receptor common gamma chain (IL2RG), is a cytokine receptor subunit that is common to the receptor complexes of at least six different interleukin receptors: IL‐2, IL‐7, IL‐9, IL‐15, and IL‐21.^101^ Lack of IL2RG function results in the near‐complete absence of T and NK lymphocytes and nonfunctional B lymphocytes, although abrogated γc cytokine‐dependent lymphocyte survival. The phenotype presents as SCID with often chronic diarrhea, a phenotype very similar to Omenn syndrome.^102^, ^103^

Combined immunodeficiency due to mutations in *DOCK2,* an activator of Rho GTPases such as RAC1 and RAC2, lead to early‐onset invasive bacterial and viral infections, lymphopenia, and various defective T‐cell, B‐cell, and NK‐cell responses. In a large international cohort, we and others showed that one of five unrelated children with defective DOCK2 developed diarrhea. *DOCK2* mutations impaired RAC1 activation in T cells and chemokine‐induced migration and actin polymerization in the T cells, B cells, and NK cells. Adding to the cellular phenotype, IFN‐α and IFN‐λ production by peripheral‐blood mononuclear cells was diminished after viral infection.^104^ Impaired T‐cell activation may account for the immune dysregulation in DOCK2 deficiency, leading to bowel inflammation.

ZAP70 is a membrane protein found on the surface of T and NK cells. It is part of the T‐cell receptor signaling cascade, crucial in the context of TCR signaling. ZAP70 deficiency, characterized by CD4 and CD8 T‐cell deficiency due to defective T‐cell receptor signaling, can present with IBD as well,^105^ potentially due to the dysregulation of T cell‐mediated immune processes.^105^

ORAI1 and STIM1 form a complex that is vital to maintain cytoplasmic‐endoplasmic reticulum calcium homeostasis of cells and is particularly important in the context of Ca^2+^‐dependent T‐cell activation.^106^ Patients with deficiency in ORAI1 or STIM1 present with variable expression of CID that is characterized by severe T‐cell activation defects, with GI manifestations previously reported in ORAI1 deficiency. These findings illustrate that impaired calcium signaling can result in gut inflammation through reduced number of Treg cells and/or aberrant T‐cell thymic selection.^107^

Patients suffering from DNA repair defects have been sporadically reported to present with IBD. This is an interesting observation as a previously reported mouse model of IBD arising in knockout of DNA repair genes has been published.^108^ However, there is currently insufficient reports of the prevalence of IBD in DNA repair defects. Among these are reports on a patient suffering from Bloom syndrome with ulcerative colitis^109^ and VEO‐IBD patient with mutation in *ZBTB24*.^110^ To date, both these report lack direct conclusion about the molecular mechanism, but highlighted chromosomal stability as one of the influential factors of IBD pathogenesis. One could hypothesize that as DNA repair is important in the context of T‐ and B‐cell maturation through V(D)J recombination, the development of IBD may be pinpointed toward lack of immune regulation.

Defects of telomere maintenance, exemplified by mutation in *DKC1* and *RTEL1* underlie dyskeratosis congenital myelodysplasia which can present with IBD.^111^, ^112^, ^113^, ^114^ In these cases, manifestation of GI inflammation can be one of the first presenting symptoms as reviewed by Jonassaint et al.^115^ They proposed that the onset of GI inflammation is due to defective epithelial barrier function as they found that these patients present with extensive apoptosis in the intestinal mucosa, potentially resulting in the breach of the epithelium and unprecedented activation of the gut immune system. However, it is likely that the T‐cell deficiency has an additional pathogenic role in the onset of bowel inflammation in these diseases.

The underlying causes of the systemic autoimmune disease in ITCH deficiency caused by defects in the *ITCH* gene are still elusive. ITCH deficiency is characterized by dysmorphic features, failure to thrive, hepatomegaly, splenomegaly, and delayed motor development,^116^ similar to the phenotype of *Itch* ^−/−^ mice.^117^ To date, two out of ten patients with ITCH deficiency have been described as developing autoimmune enteropathy and chronic diarrhea, with lymphocytic inflammation of the lamina propria.^116^ As a ubiquitin ligase, ITCH attaches ubiquitin to substrate proteins and marks them for lysosomal degradation.^118^ Ubiquitination is a key component of multiple signaling cascades of the immune system, including TCR downregulation. The exact molecular mechanism behind the systemic autoimmune disease in ITCH deficiency are unclear; however, it might be due to similar mechanics as dysfunction of other E3 ligases Cbl‐b and GRAIL, which catalyze the final step of ubiquitin attachment, that can lead to indiscriminate T‐cell activation and loss of tolerance to self‐antigens.^119^, ^120^

### 2.6 Defects in Tregs or IL10 signaling

The discovery of biallelic LOF mutations in the *IL‐10 receptor* genes presenting with bowel inflammation as the main phenotype have highlighted the pivotal role of IL‐10, and IL‐10 in Treg cell function especially in the gut. Defects in the IL‐10 receptor genes *IL10RA* and *IL10RB* and *IL‐10* itself lead to early‐onset enterocolitis involving hyperinflammatory immune responses in the intestine due to abrogated interleukin‐10‐induced signaling and therefore improper function of regulatory T cells.^26^ Similarly, immune defects abrogating proper T_reg_ function can lead to bowel inflammation as well. Immune dysregulation, polyendocrinopathy, and enteropathy (IPEX) is caused by mutations in the *FOXP3* gene, a master regulator of the development and function of Tregs. In IPEX, the lack of or mutant FOXP3 protein causes abnormal T_reg_ function, which causes systemic autoimmunity and severe enteropathy associated with eosinophilic inflammation.^121^ Mutations in *CD25* encoding IL2RA, a protein constituting the high affinity IL‐2 receptor results in an IPEX‐like syndrome. The patients exhibited defective IL‐10 expression from CD4 lymphocytes, highlighting the importance of IL‐2 in IL‐10 production, and the priming of T_reg_ for immunosuppressive functions.^122^

CTLA‐4 is an essential effector component of T_reg_ cells that is required for their suppressive function.^123^ Therefore, CTLA‐4 is a critical inhibitory checkpoint of immune responses. The crucial role of the negative regulation by CLTA‐4 is illustrated by the lethal autoimmunity developed by Ctla4‐deficient mice.^124^ CTLA‐4 resides in intracellular vesicles on T_reg_ and is released and mobilized to the cell surface after TCR stimulation, where it works as an “off” switch when bound to either CD80 or CD86 on the surface of antigen‐presenting cells.^125^, ^126^ CTLA‐4 haploinsufficiency or impaired ligand binding results in a complex syndrome presenting with features of both autoimmunity and immunodeficiency.^127^

Patients with CTLA‐4 haploinsufficiency develop autoimmune thrombocytopenias and abnormal lymphocytic infiltration of non‐lymphoid organs, including the lungs, brain, and GI tract, resulting in enteropathy.^128^ CTLA‐4 haploinsufficiency has been observed to have incomplete penetrance. However, as the age of studied patients ranges from 7 to 40, currently healthy mutation carriers may develop disease later on in life. Indeed, autoimmune features (psoriasis, type 1 diabetes, and prolonged episodes of diarrhea) are evident in carriers previously classified as healthy. Patients with biallelic mutations in the *LRBA* gene present with a phenotype clinically resembling CHAI disease, but with recessive inheritance.^129^ LRBA plays an immunoregulatory role in the expression, function, and trafficking of CTLA‐4 from the intracellular vesicles to the cell surface. In fact, patients with *LRBA* mutations show CTLA‐4 loss and immune dysregulation^125^ and can present with VEO‐IBD.^32^, ^130^

A considerable fraction of patients with Wiskott‐Aldrich syndrome (WAS) can develop IBD or IBD‐like gastroenterocolitis. WASP is expressed in hematopoietic cells and plays essential roles in signal transduction, cell‐cell interactions, cell movement, and cell division. The mechanisms driving gut abnormalities in patients with *WAS* mutations most likely have a broader etiology and, like for LAD1 patients, are not restricted to neutrophil defects. Wasp‐deficient mice develop chronic colitis associated with colon crypt hyperplasia and the presence of mixed lymphocytic and neutrophilic infiltrate within the lamina propria.^131^ Defects in T_regs_ and expansion of autoreactive B cells are likely the main drivers of IBD/IBD‐like colitis in WAS patients. Impaired regulatory T cells may also affect the microbiota, leading to dysbiosis that may contribute to colitis development.^132^

The STAT family of transcription factors plays a critical role in mediating responses to cytokines, thereby influencing and initiating cell activation, survival, and proliferation.^133^ Autosomal dominant GOF mutations in *STAT3* result in infantile‐onset multisystem autoimmune disease. Common manifestations include insulin‐dependent diabetes mellitus and autoimmune enteropathy, or celiac disease, and autoimmune hematologic disorders.^134^ It is postulated that GOF mutations in *STAT3* lead to autoimmunity, and thereby autoimmune enterocolitis through impairing the development of regulatory T‐cells and promoting the expansion and activation of Th17 cells.^135^, ^136^

Lack of T_regs_ in a combined immunodeficiency due to *MALT1* mutations (compound heterozygous splice acceptor and de novo deletion) has been recently described in a male infant who developed generalized rash, intestinal inflammation, and severe infections including persistent cytomegalovirus.^137^ MALT1 is a paracaspase with a central role in the activation of lymphocytes and other immune cells including myeloid cells, mast cells, and NK cells. MALT1 activity is required not only for the immune response, but also for the development of natural T_reg_ cells that keep the immune response in check and is an essential regulator for NF‐κB activation.^138^ Its inhibition attenuated symptoms of dextran sodium sulfate‐induced colitis in mice reducing activation of NF‐κB and NLRP3 inflammasome in macrophages.^139^ MALT1‐deficient patients fail to generate memory and Treg cells and develop hypogammaglobulinemia, due to impaired NF‐κB signaling in lymphocytes resulting in immune dysregulation.

Integrity of the TCR/CD3 complex is vital for proper T‐cell maturation and function. Mutations in T‐cell surface glycoprotein CD3 gamma chain (CD3γ) abrogate the integrity of the complex and result in autoimmunity, accompanied by IBD, due to T‐cell phenotypic and functional defects, especially in T_reg._ 140 Therefore, it is postulated that the pathomechanism of IBD in CD3G deficiency stems from the dysregulation due to reduced T_reg_ function.^140^

Mutations in the *IL21* gene, a critical regulator of STAT1, STAT3, and STAT5 signaling^141^ cause early‐onset IBD and common variable immunodeficiency‐like disease.^31^ In the context of IL‐21 deficiency, the IBD phenotype could be explained by the lack of anti‐inflammatory action of I‐L21 in inducing IL‐10 production through a STAT3‐mediated signaling axis. However, this might not be the only mechanism as IL‐21R deficient patients have not been reported to develop IBD. More patients need to be identified prior to a conclusive genotype‐phenotype correlation.^141^

### 2.7 Systemic autoinflammatory diseases and IBD

Systemic autoinflammatory diseases denote a group of immune dysregulatory conditions that usually present in early childhood with fever and disease‐specific patterns of inflammation. Studying the gene defects underlying the recurrent inflammatory episodes has revealed key immune pathways underlying persistent inflammation such as excessive IL‐1 signaling, constitutive NF‐κB activation, and chronic type I IFN signaling.^142^ VEO‐IBD has been described as an accompanying phenotype in a number of systemic autoinflammatory diseases. Many of the exact causal mechanisms are still postulated, but it is likely that molecular defects underlying IBD in these autoinflammatory conditions disrupt the delicate homeostasis of immune cells, epithelial cells, and the microbiota in the gut by chronically activating proinflammatory pathways and cell types. The importance of such intrinsic innate signaling systems such as IL‐1β signaling in the pathogenesis of IBD is illustrated by the fact that inhibiting IL‐1β signaling can induce complete or partial elevation of symptoms in patients, including the remission of the VEO‐IBD phenotype.^143^, ^144^

Mevalonate kinase deficiency due to pathogenic mutations in the *MVK* gene presents with hyper IgD syndrome (HIDS), as well as polyarthralgia or nonerosive arthritis of large joints, cervical lymphadenopathy, abdominal pain, vomiting, diarrhea, and variable skin lesions, including maculopapular, urticarial, nodular, and purpuric rashes.^143^ LOF mutations in *MVK,* encoding a key enzyme in the cholesterol synthesis pathway, impair the enzymatic activity and lead to a shortage of farnesyl pyrophosphate and geranylgeranyl pyrophosphate, intermediates for isoprenoid synthesis and substrates used for protein prenylation.^145^, ^146^ Flares in HIDS are thought to be the result of uncontrolled release of IL‐1β as a consequence of insufficient geranylgeranyl pyrophosphate generation.^147^

*PLCG2* encodes phospholipase Cγ2 (PLCγ2), an enzyme responsible for ligand‐mediated signaling in cells of the hematopoietic system through IP3 and DAG, and plays a key role in the regulation of immune responses. Patients with GOF mutations in *PLCG2* develop autoinflammation and PLCγ2‐associated antibody deficiency and immune dysregulation (APLAID). APLAID presents with recurrent blistering skin lesions, bronchiolitis, arthralgia, ocular inflammation, enterocolitis, absence of autoantibodies, and mild immunodeficiency, with a decrease in circulating IgM and IgA antibodies, decreased numbers of class‐switched memory B cells, and decreased numbers of Natural Killer T (NKT) cells.^148^ The phenotype in APLAID is thought to be the consequence of the GOF mutations that create an extra phosphorylation site which enhances activation *PLCG2* by compromised (although not completely abrogated) autoinhibition of PLCG2 activity. Intriguingly, *PLCG2* genomic deletions in individuals present with a distinct inflammatory disease manifested by cold‐induced urticaria and immune dysregulation including features of both immunodeficiency and autoimmunity, called PLAID. The PLAID‐associated genomic deletions disrupt the cSH2 domain of PLCγ2, resulting in constitutive phospholipase activity. Despite the constitutively active enzymatic activity, PLAID patients have reduced PLCγ2‐mediated signal transduction at physiologic temperatures most likely as a result of a negative feedback caused by constitutive activation.

Mutations in two of the genes encoding for the inflammasome components NLRC4 and MEFV can cause monogenic autoinflammatory diseases that can present with IBD. Recessive and postulated autosomal dominant mutations in *MEFV*, a gene encoding the intracellular sensor pyrin/marenostrin, cause familial Mediterranean fever (FMF). FMF flares include fever, generalized peritonitis, and less frequently nonerosive oligoarthritis, and can include colitis.^149^, ^150^ MEFV has been implicated in multiple cellular and vital immune functions such as the assembly, intracellular danger sensing and induction of inflammation by the inflammasome, intracellular danger signal sensing, apoptosis, and autophagy in granulocytes and monocytes.^151^ Although the concrete link between the FMF phenotype with colitis and MEFV is still to be understood, it is clear that mutations in *MEFV* result in the enhanced and extended inflammatory response to some of the innocuous factors that are tolerated well and handled efficiently by the normal immune system. Activating heterozygous mutations in *NLRC4* have been reported to cause recurrent fevers and severe systemic inflammation, similar to macrophage activation syndrome (MAS). To date, three of 4 reported patients developed enterocolitis.^152^, ^153^ NLRC4, a member of cytoplasmic NOD‐like receptors, is involved in detection of pathogen‐associated molecular patterns and initiate inflammatory responses by recruiting and proteolytically activating caspase‐1 within the inflammasome upon stimulation. Mutant NLRC4 causes constitutive IL‐1 and IL‐18 family cytokine production, macrophage activation, and increased cell death. Patient macrophages are polarized toward pyroptosis and exhibit abnormal staining for inflammasome components.

Heterozygous germline mutations in *TNFAIP3* cause a Behçet's‐like disease, characterized by early‐onset systemic inflammation, arthralgia/arthritis, oral/genital ulcers, and ocular inflammation described in six unrelated families.^154^ *TNFAIP3* encodes the NF‐κB regulatory protein A20 which is a potent inhibitor of the NF‐κB signaling pathway via its deubiquitinase activity. *TNFAIP3* mutant patient‐derived lymphocytes show increased degradation of IκBα and nuclear translocation of the NF‐κB p65 subunit, together with increased expression of NF‐κB‐mediated proinflammatory cytokines. In these lymphocytes, TNF stimulation leads to defective removal of Lys63‐linked ubiquitin from TRAF6, NEMO, and RIP1.^154^

LOF mutations in the gene encoding CASP8, a protease that initiates apoptosis and regulates immune responses have been described very recently to cause infant‐onset IBD.^155^ Previously, patients with *CASP8* mutations have been shown to present with autoimmune lymphoproliferative syndrome‐like (ALPS) like disorder.^156^ In contrast, the novel report shows patients with previously undocumented mutations in *CASP8* presenting with severe VEO‐IBD as the main clinical manifestation. The patient lymphocytes exhibited defective T‐ and B‐cell maturation proliferation and activation, as well as impaired inflammasome activation and defective epithelial cell death responses. These findings highlight the critical role of CASP8 in non‐apoptotic functions, especially in maintaining intestinal immune homeostasis.

Abnormal nucleic acids generated during viral replication is one of the main triggers for antiviral immunity. Concomitantly, mutations disrupting nucleic acid metabolism can lead to autoinflammatory disorders. SKIV2L is an RNA helicase and is an important negative regulator of the RIG‐I‐like receptor (RLR)‐mediated antiviral response. Mutations in *SKIV2L* cause THES, characterized by chronic diarrhea, liver disease, hair abnormalities, and high mortality in early childhood due to severe infection or liver cirrhosis.^157^, ^158^ It has been shown that the unfolded protein response (UPR), which generates endogenous RLR ligands through IRE‐1 endonuclease cleavage of cellular RNAs, triggers type I interferon (IFN) production in SKIV2L‐depleted cells.^159^ Intriguingly, THES can be caused by mutations in *TTC37* 86, 87 where, in contrast to SKIV2L, in vitro assays do not propose a role in interferon signaling. This suggests that most of the features of THES are most likely the consequence of a loss of cytosolic RNA exosome function in RNA turnover, instead of aberrant interferon response that is apparently specific to SKIV2L deficiency.

Intronic mutations in *DNA Polymerase Alpha 1* (*POLA1*) cause X‐linker reticulate pigmentary disorder including early‐onset IBD. *POLA1* encodes the catalytic subunit of DNA polymerase and is vital component of the DNA replication machinery. The polymerase A complex synthesizes RNA:DNA primers which initiate the production of Okazaki fragments. Mutations in *POLA1* affect the expression of DNA polymerase‐α, leading to aberrant synthesis of RNA:DNA primers in cells, thereby inducing type 1 IFN.^160^, ^161^

Patients with mutations in *ADAM17* present with early‐onset pustular dermatitis, short and broken hair, paronychia, frequent cutaneous bacterial infections, cardiomyopathy, and early‐onset diarrhea.^162^ In a study of two related patients, patient‐derived PBMCs showed high levels of lipopolysaccharide‐induced production of interleukin‐1β and interleukin‐6 but impaired release of TNF‐α.^162^ ADAM17 plays a role in the processing of other cell surface proteins, including a TNF receptor, the L‐selectin adhesion molecule, and transforming growth factor‐alpha (TGF‐α).^163^ Although direct links between the patient's phenotype and ADAM17 defects is still elusive, lack of TNF‐α is considered partly responsible for the increased susceptibility to infection and development of cardiomyopathy, and as *Adam17* knockout mice present with impaired epithelial cell maturation in multiple organs, the lack of proper epithelial barrier could be postulated to stem the IBD phenotype.

### 2.8 Complement deficiencies

The complement system is made up of a large number of distinct plasma proteins and autologous cell surface proteins that react with one another to mainly opsonize pathogens and induce a series of inflammatory responses, initiating the adaptive inflammatory response. Deficiencies in complement proteins mostly manifest as recurrent bacterial infections due to defective bacterial clearance and autoimmunity such as systemic lupus erythematosus. However, multiple cases of complement deficiency presenting with IBD or IBD‐like symptoms have been sporadically reported,^164^ pointing to a possible role of complement pathway in IBD pathogenesis. The potential pathomechanism of IBD pathogenesis in complement deficiencies has been hypothetically directed toward defective bacterial clearance and potential defective epithelial defense against complement attack. In this case, the interplay between the microbiota and immune system is further highlighted as the manifestation of bowel inflammation is present in all patients.

The identification of MASP2 deficiency highlighted the potentially vital role of proper activation of the complement system in colitis.^165^ In one patient, homozygous mutation in the *MASP2* gene caused defective activation of the complement system through the mannan‐binding lectin (MBL) pathway, and resulted in a presentation of ulcerative colitis and later on erythema multiforme bullosum. Numerous polymorphisms in *MASP2* that causes lack of MBL pathway activation have been identified,^166^ but no further reports of IBD have been described. Therefore, MASP2 might be a modulator of IBD pathogenesis and that requires further triggers to result in an IBD presentation.

Ficolin 3 deficiency was first reported in a patient with immunodeficiency and recurrent infections, clinical manifestations that are in line with complementopathies. In a report by Shlapbach et al^167^, 2 patients with congenital FCN3 deficiency suffered from severe, potentially fatal necrotizing enterocolitis that they postulate was due to defective control of intestinal microbiota leading to local inflammation.

In 2017, we and others have identified biallelic LOF mutations in *CD55* encoding for the protein decay accelerating factor (DAF) in patients with severe early‐onset protein‐losing enteropathy.^168^ CD55 is a complement regulatory binding protein present on autologous cells that acts to prevent the activation of the complement cascade on cell surfaces. It does so by binding to C3b and C4b, two complement convertases and silences their activity. To date, a total of 18 patients have been described in the literature to have mutations in *CD55* affected with protein‐losing enteropathy and of these, 6 develop bowel inflammation with histologically proven lymphocytic infiltrates in the mucosa or mucosal ulcers. However, the extent of the inflammation is not as severe as in other EO‐IBD patients. The origin of the inflammation is still unclear, but we propose 2 potential pathomechanisms: dysregulation of immunoregulatory T cells, similar to observation made in mouse models on the role of CD55 on T_reg_ homeostasis,^169^ and epithelial and/or endothelial barrier damage due to complement activation. Interestingly, some patients develop thrombotic events, a clinical manifestation of many complementopathies. Patients responded well to the eculizumab treatment,^170^ with immediate effects seen in the GI protein loss clinical manifestation. However, more data need to be obtained to see if eculizumab proves to be efficacious in relieving bowel inflammation in CD55‐deficient patients.

### 2.9 Other gene defects

IBD or an IBD‐like phenotype have been described in diseases with no well‐defined plausible mechanisms, or in diseases where well‐defined molecular mechanisms exist but the underlying cause of IBD is still elusive.

Defects in HPS1, HPS4, HPS6 genes that underlie Hermansky‐Pudlak syndrome (HPS), can present with colitis.^171^, ^172^ Patients presents with the triad of oculocutaneous tyrosinase‐positive albinism, prolonged bleeding time secondary to platelet storage pool defect and ceroid depositions within the reticuloendothelial system. Reportedly, some patients develop GI complications related to chronic granulomatous colitis, enterocolitis, and extensive granulomatous perianal disease. Although some evidence suggests that an abnormality of lysosomal function may be responsible for the development of the disease, the underlying molecular mechanisms are still unclear. More intriguingly, mutations in *HPS3*,* HPS5,* and *HPS7* cause HPS that do not present with IBD.

PEPD encodes a member of the peptidase family with an important role in recycling of proline and might be rate limiting for the production of collagen.^173^ Individuals with mutations in PEPD develop prolidase deficiency, characterized by lack of peptidase activity, skin ulcers, mental retardation, and recurrent infections. Patients may have splenomegaly, and in some cases, hepatosplenomegaly. Diarrhea, vomiting, and dehydration may also occur.^174^, ^175^ Pathogenic mutations in *PEPD* lead to reduction or loss of prolidase activity which may contribute to the multifactorial clinical presentation. Since phenotype, age of onset, and clinical course of prolidase deficiency are very variable even within the same family, and the number of molecularly characterized patients is very small, it is still difficult to define a genotype‐phenotype relationship for this disease.^173^

Complex dysregulation of transforming growth factor beta as a result of autosomal dominant mutations in *TGFBR1* and *TGFBR2* (Loeys‐Dietz syndrome) cause a syndrome with a variety of phenotypes including skeletal involvement, arterial abnormalities and immunological abnormalities, IBD, and encelopathy.^176^ Recently, biallelic LOF mutations in the *TGFB1* gene encoding TGF‐β1 have been described in patients with central nervous system disease including epilepsy, brain atrophy, and posterior leukoencephalopathy, and severe VEO‐IBD.^177^ The mutations in *TGFB1* seemingly impaired the bioavailability of TGF‐β1. Although the exact mechanisms of how impaired TGF‐β signaling leads to IBD is yet to be determined, these findings suggest a pivotal role in of TGF‐β immune function, especially in intestinal immune homeostasis.

Defective adaptation to hyperosmotic stress in lymphocytes recently emerged as one of the novel mechanisms underlying IEI and IBD. A single male with de novo Nuclear Factor of Activated T Cells 5 (NFAT5) haploinsufficiency presented with autoimmune enterocolopathy, unexplained infections, and bowel inflammation. Further examination revealed IgG subclass deficiency, impaired antigen‐induced lymphocyte proliferation, reduced cytokine production by CD8^+^ T lymphocytes, and decreased numbers of NK cells.^178^ NFAT5 is a transcription factor protein that is activated in response to osmotic stress. In NFAT5‐deficient patients, regulation of immune cell function and cellular adaptation to hyperosmotic stress is abrogated, leading to the phenotype.

Dysregulation of mitochondrial integrity and increase in cellular stress have been recently identified as a cause of severe T‐, B‐, and NK‐cell lymphopenia presenting with VEO‐IBD. Two patients, one with homozygous and one with compound heterozygous mutations in ankyrin repeat and zinc‐finger domain‐containing 1 (*ANKZF1*) developed severe bowel inflammation, severe ulcerative skin lesions, and T‐, B‐, and NK‐cell lymphopenia. The suspected causal gene, *ANKZF1* has a role in mitochondrial response to cellular stress. As a consequence of mutations in *ANKZF1*, mitochondrial respiration is impaired resulting in increased apoptosis in patient lymphocytes.^179^

## 3 GENOMICS AND ITS INFLUENCE ON THERAPEUTIC GUIDELINES FOR VEO‐IBD PATIENTS

VEO‐IBD patients make interesting clinical cases as this group of rare diseases often comes without a clear‐cut clinical decision‐making scheme as they often present with multi‐organ involvement that requires intervention from different clinicians. Treatment of VEO‐IBD patients does not differ from adult‐onset IBD patients in principle, in that the end result is to induce and maintain remission. These patients receive a standard care therapy, which frequently involves a combination aminosalicylates, corticosteroids, immunomodulators, antibiotics, and/or biologics. These medications aim to control intestinal inflammation by dampening the immune system. However, due to the heterogeneous clinical response of VEO‐IBD patients to immunomodulatory drugs, it is often difficult to prescribe a clinical guideline for treatment.

In the more severe cases, bowel resections may be performed to reduce inflammatory regions in the GI tract.

The identification of underlying genetic causes of the disease can highly influence the clinical decision making for patients with a mutation in known disease‐causing genes. For instance, hematopoetic stem cell transplantation is currently the only curative therapy for patients with IL‐10R deficiency^26^ and has been shown to result in a positive clinical outcome in some patients with LRBA deficiency.^180^ Treatment of CTLA‐4 haploinsufficiency and LRBA are prime examples of genome‐informed precision medicine, where treatment with Abatacept (CTLA‐4‐Ig) has proved to be successful in alleviating the infiltrative and autoimmune disease.^125^, ^181^

In the case of a genetic mutation in a gene that affects both the immune and epithelial barrier (for example TTC7A deficiency), HSCT did not correct for the epithelial‐intrinsic defect and enteral tolerance.^182^ This further highlights the importance of identifying underlying genetic cause of VEO‐IBD to reduce treatment‐related mortality. More research needs to be performed in order to elucidate the roles of gene defects in cell types in which they were not implicated before.

## 4 BOWEL INFLAMMATION AND THE MICROBIOME

While the link between gut inflammation and gut dysbiosis is not a novel concept, the development of culture‐independent techniques like next‐generation sequencing and metagenomics exploded the field of microbiome‐related studies. These techniques enabled the global assessment of the gut microbiota more accurately and in a more sophisticated manner.^183^, ^184^ The largest and perhaps the most ambitious initiative that has emerged in the last decade to study the changes of the human microbiome in health and disease is the NIH sponsored Human Microbiome Project (HMP).^185^ It has resulted in the publication of 5177 microbial taxonomic profiles from a population of 242 healthy adults and serves as a comprehensive database for research in this field.^186^ This project was followed up by the second phase that, in addition to phylogenic composition, aimed to analyze functional omic data including transcriptome, proteome, and metabolome. Such multi‐omic approaches with simultaneous analysis of host and microbiome proteins and metabolites aimed to better our understanding of the biology of the microbiome and sophisticated molecular mechanisms of host‐microbiota interaction.^187^ Such integrative analysis is the key feature of the future microbiome research.^188^, ^189^

While microbiota from some body sites (for example skin) is easily accessible, the GI tract is much more challenging to sample and describe. The complex structural and functional features of the human GI tract is reflected by the differences in abundance and composition of bacteria and their dynamic variations along the intestine make human microbiome studies complex.^190^ The excitement in studying the gut microbiome is not only driven by the fact that it is perhaps the most abundant and complex microbial community of the human body, but that it has also been associated with the development of wide spectrum of diseases. Indeed, numerous studies, including those that use integrative analysis of human gut microbiome and metabolome, have associated the gut microbiota with the promotion of health and development of IBD, obesity‐related inflammatory disorders, allergic diseases, and infectious diseases.^191^ Although the correlation between gut dysbiosis and IBD is well appreciated, the role of microbiome perturbations in disease development is not yet clearly defined.^192^

The role of the immune system in the preservation of healthy gut microflora is highlighted by the studies of IEI, showing that diverse pathomechanisms may underlie development of gut inflammation in immunocompromised patients. Studies of both adult and pediatric IBD showed decreased diversity of microflora in patients with CD and UC, increased numbers of mucosa‐associated aerobic and facultative‐anaerobic bacteria in colonic biopsies and perturbations in two most abundant fila—Firmicutes and Bacteroidetes.^187^, ^193^, ^194^, ^195^, ^196^ While microbiome perturbations in IBD can have a complex etiology, dysbiosis in patients with VEO‐IBD or IEI is driven primarily by the gene defects. A study of the gut microbiome in CVID patients showed significant differences in bacterial composition with dysbiosis and low alpha diversity characteristic of the patients with IBD.^197^ Interestingly, while elevated dysbiosis index was a characteristic of patients “with infections only” and “with complications” subgroups, the latter had also reduced alpha diversity of the gut microbiota. Patients with enteropathy within the “with complications” subgroup did not show any significant differences in gut microbiota. The lack of obvious differences in microbiomes between patients with or without gut pathologies in this study is difficult to explain. No systematic studies to date involving genetically characterized VEO‐IBD and IEI in patients have been conducted, and it is not clear whether these patients might develop gene defect‐specific perturbations in the gut microbiome. Given the diversity of molecular pathomechanisms underlying IBD in patients with immune defects, one could speculate that their effect on the microflora might be quite different.

To date, a variety of mechanisms explaining how changes in gut microflora may impact host immune system have been described. This topic has had substantial advancements, highlighting some exciting examples of bacteria‐derived metabolite involvement. Playing a pivotal role in maintaining organismal homeostasis and stable physiology, microbiota produce, degrade, and modulate a large number of small molecules—metabolites, complementing the host metabolic capacities. Another important function of this bacteria‐modified metabolic network is communication with the host.^198^ Even in a healthy state of intact gut epithelial integrity, many bacterial metabolites are absorbed, drain into the portal vein and can be detected in the periphery if they are not metabolized in the liver. Three main mechanisms of how bacterial metabolites impact the immune system have been described: (a) through binding to the specific cell surface receptors, (b) inflammasome‐forming intracellular receptors, and (c) antigen presentation.

Short‐chain fatty acids (SCFAs), tryptophan metabolites, and retinoic acid (RA) are the most illustrious examples of metabolites that are involved in various aspects of immune cell regulation, development, and differentiation of activation‐specific G‐protein‐coupled‐receptors. Downstream signaling through these receptors is responsible for T_reg_ expansion and differentiation, decrease of proinflammatory Th17 cells, changes in neutrophil, and lymphocytes chemotaxis, and hematopoiesis of dendritic cells from bone marrow. SCFA such as butyrate and propionate are known to act as histone deacetylase (HDAC) inhibitors. Butyrate suppresses proinflammatory effectors in lamina propria macrophages and differentiation of dendritic cells from bone marrow stem cells via HDAC inhibition, resulting in hyporesponsivness to commensals. In addition, SCFAs also regulate cytokine expression in T cells and generation of regulatory T_reg_ through HDAC inhibition.^199^

Some commensal microorganisms like *Lactobacilli* use tryptophan as an energy source to produce ligands of the aryl hydrocarbon receptor (AhR), such as the metabolite indole‐3‐aldehyde. AhR is a ligand‐activated transcription factor critically important to the organogenesis of intestinal lymphoid follicles (ILFs). AhR is also expressed by immune cells, including RORγt^+^ group 3 innate lymphoid cells (ILC3s) that are involved in ILF genesis, and AhR expression on ILC3s is functionally required for their expansion. AhR‐induced IL‐22 production by ILCs drives the secretion of the anti‐microbial peptides lipocalin‐2, S100A8, and S100A9, which protect from pathogenic infection by *Candida albicans*. In addition to its role in the function of ILCs, AhR was also found to be necessary for the maintenance of the epithelial barrier and the homeostasis of intraepithelial lymphocytes (IELs).^198^

Retinoic acid (RA) signaling has been shown to be important in the myeloid compartment. Specific subsets of intestinal DCs and macrophages constitutively produce RA and induce T_reg_ development through RA receptors. In addition, signaling downstream RA receptors induce expression of gut homing receptors on activated T and B cells and enhanced induction of immunoglobulin A (IgA) by B cells.^200^

The role of vitamins in maintaining T_regs_, as well as a number of lymphocytes and NK‐cell activity, has also been established.^198^ In this case, the effect is mediated through specific receptors broadly expressed on various subsets of immune cells.

The modulation of inflammasome signaling by bacteria‐derived metabolites is another distinct mechanism involved in modulation of host immunity. Recent studies implicated several low‐molecular‐weight compounds associated with metabolism, not immunity, in regulation of NLRP3 and NLRP6 activation.^201^ In a recent study, it has been shown that microbial metabolites taurine, histamine, and spermine modulate NLRP6 inflammasome signaling, secretion of IL‐18, and production of anti‐microbial peptides shaping the host‐microbiome interface.^202^

The discovery of bacteria‐specific vitamin B metabolites recognized as antigens by mucosa‐associated invariant T (MAIT) cells revealed yet another mechanism of host‐microbiome interaction and provides an important hint as to how our immune system may sense and control the microbiome.^203^ Protective role of MAIT cells upon bacterial infection and their role in autoimmune diseases such as multiple sclerosis and IBD makes these cell attractive targets for clinical interventions. Despite a huge interest in these unique T‐cell subsets, their role in disease pathogenesis is still not clear complicating their therapeutic implementation. In addition, only few bacteria‐derived molecules have been identified to date with agonistic or antagonistic effect on MAIT cells. Interestingly, a novel heterogeneous population of T cells has been recently identified. These cells recognize endogenous metabolites of unknown structure presented by MHC class I‐related molecule 1 (MR1) the same molecule that present bacterial metabolites to MAIT cells.^204^ The spectrum of stimulatory antigens and molecular mechanism of antigen presentation to MAIT and other MR1‐restricted T cells are still the subject of active research.

Increasing numbers of studies with mouse models of colitis show protective effect of microbiota transfer. Fecal microbiota transplantation has been described as safe and promising treatment for IBD, with unexplained variable efficacy.^205^

Studies have shown that colitis in Nlrp12‐deficient mice can be reversed equally by treatment with antibodies targeting inflammatory cytokines and by the administration of beneficial commensal isolates. Such contributions of the microbiome to the development of gut inflammation reveal a feed‐forward loop in which a genetic defect promotes dysbiosis that further contributes to the development of gut inflammation.^206^ Studies in Il10 and Nlrp6‐deficient mice show that adaptive and innate immunity defects may have different contributions to the development of spontaneous colitis, inflammation, and colonization by a specific pathobionts.^207^ Overall, studies of gut microbiome in respect to metabolite composition and dynamics provide a basis for the targeted metabolomic intervention and treatment or prevention of dysbiosis‐driven diseases. This approach is exemplified by the well‐documented beneficial effects of short‐chain fatty acid butyrate, synthesized from non‐absorbed carbohydrate by gut microbiota. A variety of approaches, including high‐fiber diet, butyrate‐producing bacteria, or coated tablets are currently in use for the butyrate‐based treatment of IBD. Classical pro‐ and prebiotic‐based therapies exhibit limited efficacy due to certain caveats such as colonization resistance and inter‐individual variation in microbial composition. Recently, a novel personalized therapeutic approach based on supplementation of the host with metabolites downstream of the microbiome which can act directly on host‐related metabolic pathways has been suggested.^208^ The nature and efficacy of such potentially bioactive metabolites to be used for the therapy require further exploration.

## 5 ORGANOIDS

Although single gene defects affecting major immune pathways have been investigated in detail, little is known about how mutations of VEO‐IBD‐associated genes are involved in epithelial barrier function or the homeostasis of the host and microbiome. As the interplay between immune cells, gut epithelial barriers, and the gut microbiota represents a central axis in the onset of VEO‐IBD, it is necessary to study the diverse genetic influences of EO‐IBD‐associated genes in these three players. Gut‐derived organoid technology has been at the forefront of advancing our understanding of gut homeostasis, in particular in studying the elusive biology of the gut epithelium. This technology has allowed us to take a reductionist approach in studying specifically gut epithelial derived from any area of the gut and has been shown to be crucial to further our understanding of the biology of some VEO‐IBD genes. For example, the role of TTC7A in controlling the polarity of the gut epithelium has been shown in organoids derived from patients with TTC7A deficiency.^37^ Patient‐derived organoids provide a potential wealth of resources for personalized medicine as it allows us to perform drug screens in the context of patients’ genetic background. This was shown in the case of patients with different mutations in the *CFTR* gene, where different genetic backgrounds show varying responses to clinically available drugs, and that the phenotype seen in the organoids correlates with patients’ clinical course on various drugs.^209^ In the future, generation of biobanks with patient‐derived gut organoids with genetically defined backgrounds will allow us to advance personalized medicine for this heterogenous group of rare diseases.

## 6 THE FUTURE OF IBD GENETICS

Next‐generation sequencing (NGS) has become widely used since 2008 to investigate the genetics of IBD. Most studies that aim to elucidate the genetic component of adult IBD focus on GWAS. However, despite all efforts and recent advances, the genotype‐molecular mechanism‐phenotype link is still missing for many of the significant GWAS loci. It has been observed that although one expects GWAS signals to cluster in disease relevant pathways and genes, association signals for complex traits tend to be spread across most of the genome including in the vicinity of numerous genes without a clear connection to disease. An “omnigenic” view of diseases proposes that in contrast to Mendelian diseases, which are often caused by high impact mutations in protein‐coding regions in a few genes, complex traits and diseases are mainly driven by lower impact variants that affect a multitude of genes and pathways often outside of the primary pathways and genes involved in the respective diseases. Given the interconnected nature of cellular systems, these lower impact variations in diverse pathways converge and create the disease phenotype.^210^ The assessment of such combinatorial effect requires complex models of molecular networks and integration of multiple datasets.

## 7 WES, PANELS, AND WGS

High‐coverage exome or targeted exome (also known as panel) sequencing are routinely used to identify genetic aberrations causing early‐onset bowel inflammation. Due to its high accuracy and moderate costs, panel sequencing is preferentially used as a screening method in many institutes. A combinatorial method is to use exome or genome sequencing, but prioritize “virtual panels”—a selected list of genes—for an initial screening and later extend the scope of the analysis to novel genes. When relying on a targeted panel approach, the design of the targeted panel is a crucial step toward appropriate gene discovery and diagnosis. On one hand, by building the panel by using only genes whose link to the disease phenotype is clear, one might miss important new genes and diagnosis. Conversely, by including candidate genes (whose association to the phenotype is yet unknown), there is an increased chance of identifying variants of unknown significance. While WES conveys an advantage of looking at all known coding sequence and can be combined with a panel approach, incidental findings and variants of unknown significance are much higher.

The percentage of patients with genetically diagnosed VEO‐IBD varies between centers and cohorts and ranges from 5%‐31% depending the composition of the cohort.^211^, ^212^, ^213^, ^214^, ^215^, ^216^ The reasons behind the missing heritability are multifactorial. Firstly, we can hypothesize that not all VEO‐IBD patients have a monogenic defect, but rather develop bowel inflammation a result of multiple genetic aberrations. In these cases, proving causality is a challenge. Secondly, inherent technical difficulties of exome sequencing, namely the challenge to detect structural variants such as insertions and deletions, copy number variations, inversions, and deletions could result in missing crucial genetic diagnoses. Naturally, as exome sequencing excludes all but coding sequences from the scope of analysis, genetic aberrations in critical regulatory regions are not detected.

Whole genome sequencing promises an unprecedented depth of study, including non‐coding regions and the analysis of copy number variations, including deletions.^217^, ^218^ Moreover, there is increasing evidence that genome sequencing might be more powerful at detecting exonic variants than whole exome sequencing.^219^ An example of applying low coverage exome sequencing to find an associative gene is the identification of ADC7Y^220^ in a study of 4280 patients at low coverage.

RNA sequencing can be used to complement genomic technologies and is routinely used for muscular disorders where it has significantly improved the rate of successful genetic diagnosis.^221^ RNA sequencing can be used to investigate transcriptomic differences between IBD and the general population, investigating alternate transcripts arising from alternative splicing and variants affecting mRNA abundance.^222^

## 8 VARIANT PRIORITIZATION AND INTERPRETATION

Along with the advent of WES and WGS, accurate variant prioritization has become even more critical to successful genetic diagnosis. WES and WGS can unravel multiple potentially causal variants and variants of unknown significance. An average exome typically contains around 30 000 variants compared to the reference genome and around 20 genes that are completely inactivated.^223^ In line with this observation, analysis of WES data focusing on rare, missense variants, and frameshift insertions and deletions often yields several (typically 20‐50) rare variants of unknown significance in a single patient. This poses a tremendous challenge to uniquely identify the causative gene variant. In the quest for the causative variants, variant interpretation, a process of connecting individual variants to disease phenotypes is essential to both reporting results to clinicians and patients and is also crucial to novel variant discovery and downstream functional research. When working under the hypothesis of a Mendelian disease, the expected causal mutations are both rare in the healthy population and severe enough to abrogate protein expression, function, or hinder initiation of vital signaling cascades.

Population‐scale variant resources such as Exome Aggregation Consortium (ExAC),^224^ the genome Aggregation Database (gnoMAD),^224^ and the 1000 Genomes project^225^ allow for filtering against population allele frequencies. Some caution should be used when relying these resources as they can contain data from yet unknown patients with various symptoms, potentially with symptoms similar to the disease of interest.

Pathogenicity prediction tools are increasingly used to assess the deleteriousness of protein‐coding variants. Some examples include protein‐based metrics such as Polymorphism Phenotyping 2 (PolyPhen2),^226^ Sorting Intolerant From Tolerant (SIFT),^227^ conservation‐based tools like Genomic Evolutionary Rate Profiling (GEPR),^228^ and integrative methods such as Combined Annotation Dependent Depletion (CADD).^229^ These tools aim to decipher the consequence of genetic variants on protein structure and function. However, these algorithms tend to exploit a single information type (conservation for example) or are restricted in scope (focusing only on missense changes, disregarding deletions and insertions). Even when different information types are used in combination (like in CADD), a lack of information is available to assess the widespread signaling and cellular perturbations of genetic aberrations and these tools are often unable to assess the effect of non‐coding, regulatory variants. Resources such as the ENCODE have facilitated the understanding of the functional and regulatory elements in the human genome, but the interpretation of non‐coding variants remains a challenge.^230^

Although filtering according to allele frequency and pathogenicity prediction provides valuable information on potential causal variants, it has become clear that the detailed characterization of the individual molecular components alone (genes, proteins, metabolites, etc.) does not suffice to truly understand the nature of (patho‐) physiological states and how to modulate them. Biomolecules do not act in isolation, but rather within an intricate and tightly coordinated machinery of complex interactions, such as protein‐protein, gene regulatory, or signaling interactions. Drawing parallels, disease phenotypes are results of a complex interplay between multiple factors. Systems biology‐based approaches have been increasingly developed and used to combine diverse types of information to assess potential causal variants and prioritize candidate variants and candidate genes. Currently, many prioritization methods and online interpretation tools exist with different approaches to data interpretation and analysis. These approaches include algorithms that use protein‐protein interactions (PPI) networks,^231^, ^232^ functional similarity networks built utilizing pathway involvement,^233^ structural variation data,^234^ exploit thorough functional annotation,^235^ or a combination of PPI based approach and phenotype similarity.^236^ In addition to available web applications and open‐source tools, professional organizations offer data interpretation and variant prioritization services.

While these algorithms have been developed to exploit diverse principles in order to prioritize variants or predict novel disease genes, there is currently no published example showcasing their validity. Algorithms and tools often fall short due to the lack of specificity or overlook important factors in a particular disease. In cases such as IBD, where the phenotype is a result of the complex interplay of multiple factors, a more tailor‐made solution could be more powerful to predict novel disease genes. An attractive avenue of prediction tools is to converge toward a contextualization‐heavy, integrative, disease‐specific variant annotation tool. In contrast to clear‐cut prioritization, this approach emphasizes annotating all potential candidates with various types of information and combines information to relate individual gene defects to known pathways and phenotypes. This approach is exemplified by the identification of causal variants in *TRIM22*, a protein which is linked to NOD2 signaling by multiple molecular networks.^71^

## 9 DATA SHARING, UNIFIED NOMENCLATURE, ONTOLOGIES

### 9.1 Toward efficient data sharing and facilitating collaborations

Research on rare diseases such as VEO‐IBD often relies on a few small pedigrees presenting with heterogeneous phenotypes. The small number of available patients and material coupled with the sheer number of potentially causal variants from NGS makes identification of causative gene defects a classical hunt for the “needle in a haystack.” In the quest for the “needle,” finding an additional patient carrying the same mutation strengthens the case of causality immensely.

Unfortunately, current variant and patient matching is hindered by the existence of small, independent datasets within the individual research groups and organizations. With no clear communication and channel for exchange, matching researchers up based on shared phenotypes and/or genotypes is simply a case of serendipity. This gap in communication and exchange is one of the main determinants of the pace of gene discovery. In order to facilitate the pace for identification of novel key players of IBD, bridging the gap between islands of research needs to be a priority.

Efforts to facilitate data and material exchange promise to bridge gaps between hospitals, specialized centers, and laboratories. Matchmaker Exchange, a project launched in 2013 addresses this crucial challenge, to facilitate the matching of cases with similar phenotypic and genotypic profiles, using standardized programming interfaces.^237^ Similar to Matchmaker Exchange, GeneMatcher, a project dedicated to enable connections between clinicians and researchers from around the world, to help unsolved exomes.^238^

### 9.2 Increasing need of high‐quality metadata

In recent years, large‐scale computation methods have been initiated to investigate the etiology of IBD. In these projects, researches often rely on public databases to provide them with data. Access to accurate genetic data is facilitated by resources such as Decipher,^239^ HGMD,^240^ OMIM^241^and ClinVar^242^ that aim to aggregate clinical data. These resources and public data repositories are still incomplete and need to be queried manually or with specifically set‐up local bioinformatics pipeline. These efforts are welcome steps toward efficient data access, but some issues with redundancy remain and variable quality and quantity of data that is still missing from these resources. A robust, unified database could be an approach worth considering.

It is becoming increasingly clear that beyond efficient access to genomic and variant information, there is a need for accurate metadata to describe clinical information not only in a genetic manner, but also phenotypically. Annotation of patients with accurate phenotype data, as well as the annotation of genes with pathways and molecular mechanisms requires standardized and objective language. Therefore, it is crucial to have a unified nomenclature and resource of disease‐causing genes annotated with the corresponding physical, molecular, and cellular phenotypes. Along with annotating patients and genes with the correct disease and ontology, intra‐institute and laboratory collaborations would benefit immensely from precise and objective descriptions of phenotypic, molecular, and genetic abnormalities.

Human Phenotype Ontology (HPO) is a phenotype vocabulary initially published in 2008.^243^, ^244^ It is a tool that enables accurate phenotyping which further facilitates efficient data and patient exchange. HPO is being increasingly adapted into everyday use as the standard to describe phenotypic abnormalities. Gene ontology (GO) on the other hand, is a computational representation of the function and localization of genes and gene products on the molecular level. Currently, the GO project has developed and constantly revised over 40 000 biological concepts and annotations.^245^, ^246^ GO provides a nomenclature to annotate gene defects with detailed molecular and mechanistic information in a unified manner. Disease ontologies aim to provide standardized, consistent, and objective descriptions of human disease terms, phenotype characteristics, and related medical vocabulary disease concepts, as well as hierarchical relationships between the disease entities themselves. Efforts are currently ongoing to translate and include diseases into ontologies such as Orphanet,^247^ Disgenet^248^, or Disease Ontology.^249^, ^250^ In addition to providing a unified nomenclature that allows clinicians and researchers to characterize patients better, the inherent network structure of ontologies such as HPO and GO allows for pairwise distance (similarity) between two terms. Consequently, pairwise similarity measures can be used to carry out complex comparisons such as testing the similarity between two patients annotated by different terms.^251^ Current ontologies have been useful in fulfilling current gap, but they are not complete. Numerous diseases and disease‐gene association are not documented, and the ontology structures are incomplete.

## 10 BEYOND GENETICS: IBDOMICS—SYSTEMS BIOLOGY AND INTEGRATIVE METHODS

One of the limitations of current approaches toward uncovering the key players in IBD pathology is that they are looking at individual contributors separately. In a case of IBD, which arises as a result of the combinatorial effect in multiple key players such as genetics, environmental factors, microbial perturbations, epigenetics, and lifestyle factors, understanding the disease cannot be tackled by studying each pathogenic component in isolation, without considering the interaction among the different “omes.” Integration of the different “omes” requires intelligently designed data integration and processing. This integrative approach, recently introduced as the IBD interactome or IBDome, calls for new concepts and tools to implement a systems biology approach toward unraveling the processes behind IBD.^252^ These tools should rely on unbiased data‐driven investigation and include strategies to reveal key drivers and pinpoint central players of inflammation and enable development of targeted therapies.

Querying and integrating multiple “omes” with bioinformatics methods enables the integration of genomic, epigenomic, transcriptomic, proteomic, metabolomics, and microbiomic data to construct a comprehensive molecular map of IBD. Although seamless data integration is yet to be available, methods to integrate various types of information and use it toward identifying key players of IBD are already underway.

Recently, Peters et al^253^ used individual networks constructed from molecular data generated from intestinal samples isolated from three populations of patients with IBD at different stages of disease, including two adult and pediatric cohorts of IBD. As a result, they developed a predictive model of the immune component of IBD that informs causal relationships among loci previously linked to IBD through GWAS using functional and regulatory annotations that relate to the cells, tissues, and pathophysiology of IBD. This network revealed potential key drivers of IBD pathogenesis. Among the key drivers were numerous known VEO‐IBD genes, but also inborn errors of immunity and new candidates that were showed to have a role in inflammatory immune response.^253^

## 11 CONCLUSION AND FUTURE PERSPECTIVES

IBD is a complex, multifactorial condition with an onset brought on by a multitude of factors that can also present in rare, Mendelian fashion. While the direct link between the more common, adult version of IBD and rare, Mendelian EO‐IBD is still elusive, we have gained tremendous understanding of different key players in immune regulation and cellular mechanisms required for immune homeostasis in the gut over the last years. The considerable fraction of inborn errors of immunity presenting with an IBD‐like phenotype highlight that all patients presenting with VEO‐IBD should be subjected to detailed genetic and immunological examination and investigation, as IBD can be an early sign of an underlying immunodeficiency. Recent advances in genomic technologies, organoid systems, as well as our increasing understanding and modeling of the interplay between the gut microbiota, immune cells, epithelial cells, and the environment promise to shed new light on to the complex molecular network behind IBD pathology. This novel understanding could allow for more efficiently identification of patient subgroups, and therefore increasingly direct treatment strategies toward personalized medicine.
